# On the selection of gantry and collimator angles for isocenter localization using Winston‐Lutz tests

**DOI:** 10.1120/jacmp.v17i1.5792

**Published:** 2016-01-08

**Authors:** Weiliang Du, Jennifer L. Johnson, Wei Jiang, Rajat J. Kudchadker

**Affiliations:** ^1^ Department of Radiation Physics The University of Texas MD Anderson Cancer Center Houston TX USA; ^2^ Department of Radiotherapy Yantai Yuhuangding Hospital, Qingdao University School of Medicine Yantai Shandong China

**Keywords:** radiation isocenter, Winston‐Lutz test, quality assurance

## Abstract

In Winston‐Lutz (WL) tests, the isocenter of a linear accelerator (linac) is determined as the intersection of radiation central axes (CAX) from multiple gantry, collimator, and couch angles. It is well known that the CAX can wobble due to mechanical imperfections of the linac. Previous studies suggested that the wobble varies with gantry and collimator angles. Therefore, the isocenter determined in the WL tests has a profound dependence on the gantry and collimator angles at which CAX are sampled. In this study, we evaluated the systematic and random errors in the isocenters determined with different CAX sampling schemes. Digital WL tests were performed on six linacs. For each WL test, 63 CAX were sampled at nine gantry angles and seven collimator angles. Subsets of these data were used to simulate the effects of various CAX sampling schemes. An isocenter was calculated from each subset of CAX and compared against the reference isocenter, which was calculated from 48 opposing CAX. The differences between the calculated isocenters and the reference isocenters ranged from 0 to 0.8 mm. The differences diminished to less than 0.2 mm when 24 or more CAX were sampled. Isocenters determined with collimator 0° were vertically lower than those determined with collimator 90° and 270°. Isocenter localization errors in the longitudinal direction (along the axis of gantry rotation) showed a strong dependence on the collimator angle selected. The errors in all directions were significantly reduced when opposing collimator angles and opposing gantry angles were employed. The isocenter localization errors were less than 0.2 mm with the common CAX sampling scheme, which used four cardinal gantry angles and two opposing collimator angles. Reproducibility studies on one linac showed that the mean and maximum variations of CAX during the WL tests were 0.053 mm and 0.30 mm, respectively. The maximal variation in the resulting isocenters was 0.068 mm if 48 CAX were used, or 0.13 mm if four CAX were used. Quantitative results from this study are useful for understanding and minimizing the isocenter uncertainty in WL tests.

PACS number: 87.56.Fc

## I. INTRODUCTION

The Winston‐Lutz (WL) test is a commonly used method to localize the isocenter of a linear accelerator (linac) in radiation therapy.^(1)^ Compared with other methods, such as room lasers and light field crosshairs, the WL test correlates the radiation fields directly with the object being irradiated. Traditional WL tests use circular or square radiation fields to image a ball‐bearing (BB) phantom on a piece of film. Images of the BB projected inside the radiation fields are obtained at selected gantry, collimator, and couch angles.^1^, ^2^, ^3^, ^4^ Through an iterative process, the BB is positioned to the center of each radiation field. The final position of the BB corresponds to the intersection of the central axes (CAX) of all sampled radiation fields, in other words, the radiation isocenter. Recently, digital WL tests have been developed in which the film is replaced with a digital flat‐panel imager such as an electronic portal imaging device (EPID).^2^, ^3^, ^4^, ^5^, ^6^, ^7^, ^8^, ^9^, ^10^ The digital images are processed with computer algorithms that locate the BB and the radiation isocenter with submillimeter precision. A new trend in digital WL tests is to keep the BB stationary during the entire test and use the BB as a reference point to localize the radiation isocenter and other image objects.^4^, ^5^, ^6^, ^7^ This concept has led to interesting applications; for example, checking the congruence between the radiation isocenter and the image centers of various on‐board imaging systems,^5^, ^10^, ^11^, ^12^, ^13^, ^14^ and analyzing the stabilities of linac mechanical components.^15^, ^16^, ^17^ The digital WL tests improve the efficiency and precision of the procedure by using digital imagers, automatic image processing, and simplified phantom positioning.

In WL tests, the radiation isocenter is determined by sampling the radiation CAX at different gantry, collimator, and couch angles. A variety of CAX sampling schemes have been reported. For the gantry angles, the majority of the reports have used four cardinal angles.^3^, ^5^, ^7^, ^9^, ^10^, ^18^ The original WL test sampled four oblique gantry angles.^(1)^ For increased efficiency, sampling of three gantry angles has also been used.^19^, ^20^ The theoretical minimal number of gantry angles required is two. On the other hand, some studies sampled eight^21^, ^22^ or more^8^, ^23^, ^24^ gantry angles to get better resolution. For the collimator angles, many studies have used one angle or the default collimator angle for conic collimators.^20^, ^25^ If a multi‐leaf collimator (MLC) was used, two opposing collimator angles were often sampled.^2^, ^3^, ^6^ Other sampling schemes employed fewer^5^, ^7^, ^14^, ^18^ or more collimator angles.^4^, ^8^, ^10^ There is no universal set of gantry and collimator angles used in WL tests. Among the possible choices, compromise is usually made between obtaining sufficient samples and reducing the test time. In this study we do not consider the selection of couch angles because (1) the couch rotation, unlike the gantry and collimator rotations, does not affect the positions of the radiation CAX, (2) the mechanical excursions of the couch can be patient‐dependent (i.e., varying with the weight of the patient and how the weight is distributed on the couch), and (3) the excursions may be corrected by realigning the couch with the lasers, which are calibrated to the radiation isocenter.^(20)^

It is known that the radiation CAX can wobble during gantry or collimator rotations. Part of the CAX wobble is systematic. For example, several studies reported that the radiation CAX moved away from the gantry when the gantry was rotated from the upright position to the lowest position by approximately 0.8 mm.^4^, ^16^, ^26^, ^27^ The causes of the CAX wobble include, but are not limited to, heavy gantry head, an aging bearing mechanism, miscalibration of MLC leaf positions, tongue‐and‐groove effect, misalignments of gantry rotation axis, collimator rotation axis, and source position. In addition, the CAX may vary with time due to beam instability.^(28)^ Given the complexity of the wobble, we hypothesize that different CAX sampling schemes in WL tests would result in different isocenter locations. However, an extensive literature search yielded few quantitative results on this subject.

The objective of the current work was to investigate the effects of gantry and collimator angle selection in WL tests on the accuracy of isocenter localization. Specifically, we studied how different sampling schemes of gantry and collimator angles affected the systematic and random errors in the resulting radiation isocenters.

## II. MATERIALS AND METHODS

### A. Digital Winston‐Lutz test

**Figure 1:**
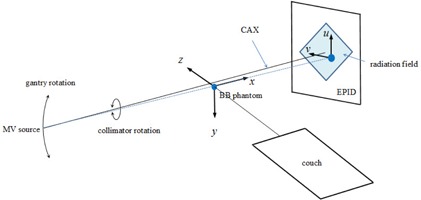
Schematic diagram of digital Winston‐Lutz test. The BB phantom is projected in a radiation field. In this example, the radiation field is a square shaped by MLC, the gantry angle is 270°, and the collimator angle is 45°.

The digital WL tests were performed on six linacs (Clinac 21EX, Varian Medical Systems, Palo Alto, CA) using a BB phantom. The BB phantom was a tungsten sphere of 6.5 mm in diameter, supported with a rod and an acrylic base block.^(11)^ Figure 1 shows a schematic diagram of the digital WL test geometry. The x‐y‐z coordinate system was static and the origin was set to the center of the BB. The BB was placed near the linac isocenter using the guidance of room lasers and served as a reference point in 3D space. The u‐v coordinate system was attached to the EPID and rotational with the gantry. The u‐v coordinates were scaled to the isocenter plane and the origin was also set to the center of the BB. At 0° gantry angle (Varian IEC 601‐2‐1 convention), the u‐v coordinates coincided with the x‐z coordinates. The six linacs in this study were designated as L1 thru L6. These linacs were installed in 2004 and had been used for patient treatments for about 10 years at the time of this study.

To acquire the WL test images, we loaded an MLC‐formed $10\times 10\;{\text{cm}}^{2}$ square field to the Varian 4DTC Treatment software with the default gantry angle (0°) and collimator angle (0°). The MLC (Millennium 120, Varian) were maintained quarterly with the vendor‐recommended procedures. The EPID (aSi‐1000; Varian) was placed at the default position, 50 cm from the isocenter. Nine gantry angles were sampled at 45° intervals: 180°, 135°, 90°, 45°, 0°, 315°, 270°, 225°, 180.1°. Starting at 180°, the gantry was rotated counterclockwise to 180.1°. The ending gantry angle 180.1° was used instead of another 180° to avoid confusion with the starting gantry angle 180°. At each gantry angle, seven collimator angles were sampled sequentially at 45° intervals: 225°, 270°, 315°, 0°, 45°, 90°, 135°. The set of seven collimator angles was sampled at the same gantry angle before moving to the next gantry angle. Due to the mechanical limitation of the linacs in this study, the 180° collimator angle was not accessible and, thus, not sampled. The gantry and collimator angles were manually entered during the beam preparation process. A 6 MV photon beam of two monitor units (MU) was used for image acquisition at each gantry angle–collimator angle combination. The dose rate was 100 MU/min. A total of 63 EPID images were acquired in one digital WL test. The pixel resolution of the EPID images scaled to the isocenter plane was 0.26 mm×0.26 mm. For each linac, one digital WL test was performed in about one hour, with the majority of time spent on manually operating the gantry and collimator rotations as part of the beam preparation.

### B. Localization of isocenter from CAX samples

The EPID images were processed offline with an in‐house MATLAB (MathWorks, Natick, MA) program. For each image, the locations of the BB center and the CAX were calculated using a Hough transform‐based algorithm that had been previously reported.^(29)^ The Hough transform algorithm was shown to localize the edges of the BB and the radiation fields with a precision better than 0.1 mm. The radiation field edges in each image were used to determine the center of the radiation field. In this study, the CAX was defined as a line passing through the source spot and the center of the radiation field. By this definition, the 3D CAX position depended on the source position, the MLC leaf position accuracy, and the tongue‐and‐groove effect, in addition to gantry‐ and collimator‐related mechanical imperfections.

For a given set of CAX samples ($\left({\text{CAX}}_{1},\;{\text{CAX}}_{2},\ldots ,{\text{CAX}}_{n}\right)$), the isocenter was calculated as the point that minimized the following cost function,

(1) $$\text{Cost}(P(x,y,z))=\sum_{i=1}^{n}d_{i}^{2}(P(x,y,z))$$

where P(x,y,z) is an arbitrary point in x‐y‐z space and $d_{i}(P)$ is the distance from point *P* to the line ${\text{CAX}}_{i}$. The resulting isocenter was the ‘best compromise’ intersection of the given set of CAX, in a least‐squares sense.

### C. Selection of gantry and collimator angles

Because the true location of the isocenter is unknown, it is impossible to derive the absolute accuracy of the calculated isocenters. Herein we assume that the accuracy can be improved by (1) using a large number of CAX to reduce the random errors, and (2) using opposing gantry angles and opposing collimator angles to reduce the systematic errors. By opposing angles, we mean angles that are 180° apart: for example, 45° and 225° are opposing angles. The use of opposing angles has been reported to compensate for systematic errors such as gantry flex and collimator misalignment.^2^, ^3^, ^6^ On the basis of this assumption, we chose the following CAX to determine a baseline or reference isocenter: gantry angle or $G=180^{\circ },135^{\circ },90^{\circ },45^{\circ },0^{\circ },315^{\circ },270^{\circ },225^{\circ }$, collimator angle or $C=225^{\circ },270^{\circ },315^{\circ },45^{\circ },90^{\circ },135^{\circ }$. A total of eight gantry angles, six collimator angles, or 48 CAX samples were used in the reference isocenter calculation. Given our data, this sampling scheme utilized the largest numbers of opposing gantry angles and opposing collimator angles in isocenter localization.

To study the effect of collimator angle selection, we varied the subsets of collimator angles while keeping the eight gantry angles ($G=180^{\circ },135^{\circ },90^{\circ },45^{\circ },0^{\circ },315^{\circ },270^{\circ },225^{\circ }$). The differences between the resulting isocenters and the reference isocenter were computed. In particular, we examined whether there were systematic errors associated with certain sampling schemes. Similarly, to study the effect of gantry angle selection, we varied the subsets of gantry angles while keeping the six collimator angles ($C=225^{\circ },270^{\circ },315^{\circ },45^{\circ },90^{\circ },135^{\circ }$).

In addition, we compared the isocenter results from other sampling schemes, including the three common ones: $3\;\text{CAX}(G=270^{\circ },\;0^{\circ },\;90^{\circ };C=0^{\circ }),4\;\text{CAX}(G=270^{\circ },\;0^{\circ },\;90^{\circ },\;180^{\circ };C=0^{\circ })$, and $8\;\text{CAX}(G=270^{\circ },\;0^{\circ },\;90^{\circ },\;180^{\circ };C=270^{\circ },\;90^{\circ })$. A complete list of all sampling schemes investigated is included in Table 1.

**Table 1:** A list of CAX sampling schemes studied. Note that the sampling scheme with 48 CAX (eight gantry angles^a^ and six collimator angles^a^) was used to generate the reference isocenter.

*Gantry Angle Sampling*		*Collimator Angle Sampling*	
*N*	*(degrees)*	*N*	*(degrees)*
		1	270
2	0, 270	1	0
2	90, 0	1	90
3	90, 0, 270	2	270, 90
4	180, 90, 0, 270	2	225, 45
4	135, 45, 315, 225	2	315, 135
8^a^	180, 135, 90, 45, 0, 315, 270, 225	4	225, 315, 45, 135
9	180, 135, 90, 45, 0, 315, 270, 225, 180.1	6^a^	225, 270, 315, 45, 90, 135
		7	225, 270, 315, 0, 45, 90, 135

### D. Reproducibility

The reproducibilities of the CAX and the resulting isocenters were evaluated on one linac (L4). First, we evaluated the CAX reproducibility without movements of the gantry or the collimator. At a fixed gantry angle (0°) and a fixed collimator angle (0°), the EPID images were acquired 20 times, each with 2 MU. The averaged CAX position was computed from the 20 CAX samples. Then the variations of these CAX samples from the averaged CAX position were calculated. Second, we evaluated the CAX reproducibility with the movements of gantry and collimator between the samples. Basically we repeated the digital WL test four times sequentially in one session. At each gantry angle‐collimator angle combination, we grouped the four sample data from these WL tests. The four CAX samples were used to compute the mean CAX and the deviation of each CAX sample. The mean, standard deviation (SD), and maximum of these CAX deviations were recorded. Because we used the BB as the reference point and the repeated WL tests were performed in one session, the reproducibility in this study was short‐term by definition. We did not address the long‐term reproducibility of the CAX.

To study the effect of CAX reproducibility on isocenter localization, an isocenter was computed from each WL test, using either the 48 CAX sampling scheme ($G=180^{\circ },135^{\circ },90^{\circ },45^{\circ },90^{\circ },135^{\circ }$; $C=225^{\circ },270^{\circ },315^{\circ },45^{\circ },90^{\circ },135^{\circ }$) or the four CAX sampling scheme $\left(G=270^{\circ },\;0^{\circ },\;90^{\circ },\;180^{\circ };\;C=0^{\circ }\right)$. Then the isocenters from the four WL tests were averaged and the deviations from the average isocenter were computed.

## III. RESULTS

**Figure 2:**
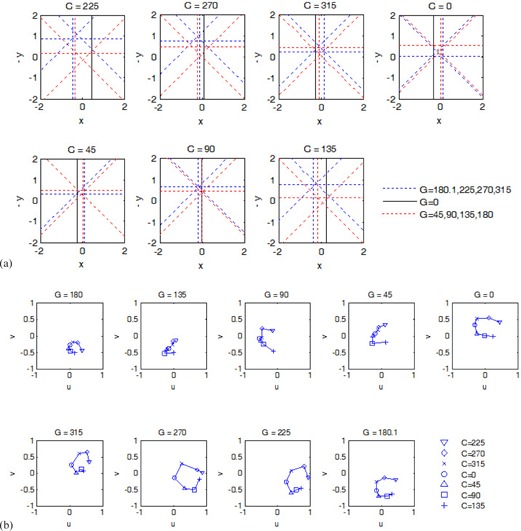
Example of CAX wobble versus gantry angle and collimator angle, measured on L6. The origin of each plot was at the center of the BB. (a) CAX viewed in the plane perpendicular to the gantry rotation axis at given collimator angles. (b) CAX viewed in the plane perpendicular to the collimator rotation axis at given gantry angles. The unit is millimeters on all axes.

Figure 2 shows an example of measured CAX locations (linac L6). The CAX plots in Fig. 2(a) simulated the conventional gantry star shot, except that the CAX were calculated from square fields instead of from narrow rectangular fields. The parallel opposing CAX are distinguished using different colors. The wobble of CAX during gantry rotation was obvious; however, the pattern of the wobble changed with different collimator angles. For example, the magnitude was smaller with C = 0° than with $C=225^{\circ }$. Figure 2(b) shows the wobble during collimator rotation in the beam's‐eye view. The wobble in the z or v direction can be seen in Fig. 2(b) but not in Fig. 2(a). The gantry sag was apparent: the CAX moved towards the –z or –v direction while the gantry angle was changed from 0° to 180° or 180.1°. The wobble during collimator rotation showed different magnitudes and yet a somewhat reproducible trend. For example, the CAX moved counterclockwise (in beam's‐eye view) as the collimator was rotated from 225° to 135°. It should be noted that the patterns in Fig. 2 were an example obtained from one linac (L6). Other linacs had different patterns of CAX wobble during gantry and collimator rotations.

**Figure 3:**
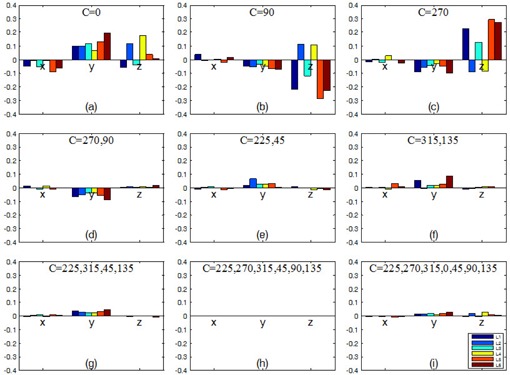
Errors in calculated isocenter locations (x, y, z coordinates in millimeters) on six linacs (L1–L6) vs. collimator angles used. The isocenter calculated with six collimator angles (h) was assumed to be the reference isocenter. In all panels (a)–(i), eight gantry angles (180°, 135°, 90°, 45°, 0°, 315°, 270°, 225°) were used.

Figure 3 shows the errors in the isocenter locations calculated with different collimator angles. Overall, the errors decreased as the number of collimator angles was increased. In addition, some systematic patterns were noticed. When only one collimator angle was used (Fig. 3(a) to (c)), the errors were up to 0.3 mm. The magnitudes of errors were generally smaller in the x direction than in the y and z directions. The errors in y were positive (pointing towards the floor) for $C=0^{\circ }$ and negative (pointing toward the ceiling) for $C=90^{\circ }$ or 270°. The errors in z did not show a consistent directional trend across the linacs; however, for each linac, the errors in z for $C=90^{\circ }$ were in the opposite direction to the counterpart errors for $C=270^{\circ }$. When two or more opposing collimator angles were used (Fig. 3(d) to (h)), the errors in z were drastically reduced to a negligible level (<0.1 mm). The errors in x were also negligible. The errors in y still showed some systematic components, depending on the collimator angles. Figure 3(i) shows that when a large number of CAX were used, the non‐opposed collimator angle, 0° in this case, did not cause a significant difference in isocenter location. The differences were less than 0.05 mm between the isocenters determined with seven versus six collimator angles.

**Figure 4:**
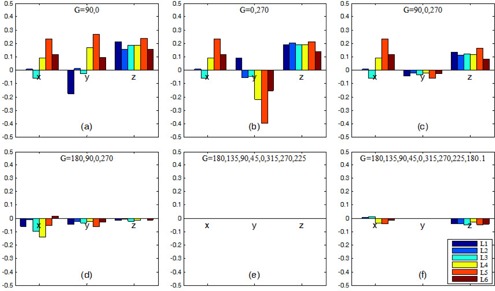
Errors in calculated isocenter locations (x, y, z coordinates in millimeters) on six linacs (L1–L6) versus gantry angles used. The isocenter with eight gantry angles (e) was assumed to be the reference isocenter. In all panels (a)–(f), six collimator angles (225°, 270°, 315°, 45°, 90°, 135°) were used.

The dependence of isocenter accuracy on gantry angle selection is shown in Fig. 4. The overall trend was that the magnitudes of the errors decreased as the number of gantry angles was increased. When only two gantry angles were used (e.g., Fig. 4(a) and (b)), the magnitudes of the errors were up to 0.4 mm. In particular, the errors in z were positive (pointing towards the gantry) across all six linacs. When three gantry angles were used (Fig. 4(c)), the paired 90°/270° gantry angles reduced the errors in the y direction significantly. The errors in x remained unchanged and the errors in z were slightly reduced. When the fourth cardinal gantry angle (180°) was added (Fig. 4(d)), the errors were reduced to <0.2 mm in all directions. In Fig. 4(f), the extra sample of 180.1° gantry angle caused a negligible (<0.1 mm) yet systematic error in the z direction.

**Figure 5:**
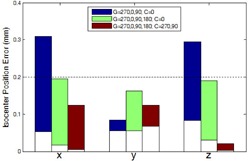
Plot of isocenter localization errors obtained with three common CAX sampling schemes. The color bars indicate the range (minimum and maximum) of data over six linacs.

Figure 5 compares the three common sampling schemes in terms of isocenter accuracy. With the first scheme, in which the gantry angle 180° was not sampled, errors in x and z were up to 0.4 mm. With the second scheme, in which the gantry angles were opposing and the collimator angle was not, the errors were reduced to 0.2 mm or less. The third scheme, using both opposing gantry angles and opposing collimator angles, reduced the errors further, especially in the z direction.

**Figure 6:**
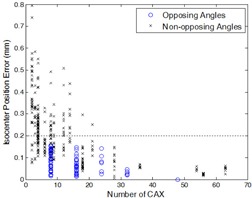
Plot of isocenter localization errors versus number of CAX used in isocenter calculation. Data with opposing gantry angles *and* opposing collimator angles are labelled with circles.

Figure 6 compares the isocenter accuracy of the 63 sampling schemes listed in Table 1. It was apparent that a larger number of CAX increased the accuracy of isocenter localization. Disregarding the angles being opposing or not, the isocenter localization errors were reduced to below 0.2 mm after the number of CAX was increased to 24 or more. With opposing gantry angles and opposing collimator angles, only eight CAX were needed to yield the 0.2 mm accuracy.

**Figure 7:**
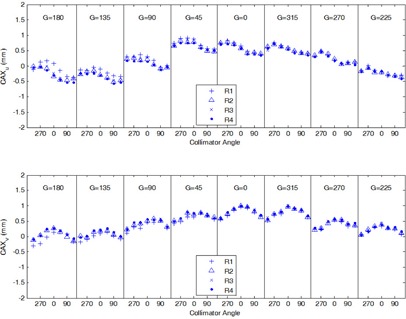
CAX reproducibility on linac L4. The digital Winston‐Lutz tests were performed in four repetitions (R1–R4). Plotted are u (top) and v (bottom) coordinates of CAX locations relative to the BB center at different gantry and collimator angles.

For the CAX reproducibility at a static gantry angle (0°) and collimator angle (0°), the mean, standard deviation, and maximum variations were 0.038 mm, 0.024 mm, and 0.087 mm, respectively. As the gantry and the collimator were changed during WL tests, the mean, standard deviation, and maximum of CAX variations were 0.053 mm, 0.045 mm, and 0.30 mm, respectively. Figure 7 shows the four CAX positions at each gantry angle‐collimator angle combination measured on L4. Notice that the largest CAX variations came from Repetition 1 (R1) and gantry angle 180°. Using the 48 CAX sampling, the mean and maximum deviations of the resulting isocenters from the average isocenter were 0.035 mm and 0.068 mm, respectively. Using the four CAX sampling, the mean and maximum deviations of the isocenter locations were 0.070 mm and 0.13 mm.

## IV. DISCUSSION

The effects of gantry and collimator angle selection on the accuracy of radiation isocenter localization were investigated in this study. In general, the random errors were reduced by sampling a large number of gantry and collimator angles; the systematic errors were reduced by using opposing gantry angles and opposing collimator angles. The magnitudes of errors in the calculated isocenters showed a significant variation ranging from 0 to 0.8 mm, depending on what gantry and collimator angles were sampled (Fig. 6). When both the gantry angles and the collimator angles were opposed, as few as eight CAX were needed to yield an isocenter accuracy of 0.2 mm (Fig. 6).

Certain systematic errors in isocenter localization are worth discussion. First, gravity may play a role in affecting the y coordinate of the isocenter position (Fig. 3). Using collimator angle 0° yielded a lower isocenter than using collimator angles 90° and 270°. This was possibly caused by the gravitational effect on the MLC leaves and carriages.^(22)^ The gravitational pull was the largest when the collimator angle was at 0° and the gantry angle was at 90° or 270°. The differences in isocenter y coordinates using collimator angle 0° (Fig. 3(a)) vs. collimator angles 90° and 270° (Fig. 3(d)) were 0.10 mm (minimum), 0.17 (mean), and 0.28 mm (maximum) across the six linacs.

Second, gantry sag in the z direction was common on all linacs in this study. If the gantry angle 180° was not sampled, the resulting isocenter had a systematic offset in z on the order of 0.2 mm (Fig. 4). This result is useful in estimating the isocenter localization errors in the WL tests or other isocenter localizing procedures that employ only two or three gantry angles ($G=0^{\circ },\;90^{\circ },\;270^{\circ }$).

Third, mechanical imperfections of the linac have different consequences on the CAX positions and the calculated isocenter position. For example, misalignment of MLC leaves with respect to the collimator rotation axis makes the CAX wobble during the collimator rotation. If CAX at opposing collimator angles are sampled, the averaged CAX position or the isocenter position is free of the effect of the MLC leaf misalignment. Similarly, opposing gantry angles and/or collimator angles minimize the MLC tongue‐and‐groove effect, misalignment of the gantry rotation axis and the collimator rotation axis, and the off‐center of the source position. On the other hand, if nonopposing gantry angles or collimator angles are used, these misalignments may propagate through the CAX wobble to the isocenter localization error. As an example, when a single collimator angle was sampled, the resulting isocenters showed potentially large errors that depended strongly on the collimator angle sampled (Fig. 3(a) to (c)).

In this study, the CAX wobble showed complex patterns during gantry and collimator rotations (Fig. 2). Consequently, wobble measured at one gantry angle or one collimator angle may not reflect the wobble at other angles. For example, the gantry star shot pattern at collimator 0° (Fig. 2(a)) alone cannot be used to characterize the size of the entire CAX wobble or the size of the isocenter sphere. In clinical practice, however, the gantry star shot is often obtained at a single collimator angle. Similarly, the collimator star shot is obtained at a single gantry angle. The WL test with MLC may be performed at a single collimator angle. The results from this study indicate that these procedures may not sample the CAX wobble adequately. Thus, the isocenters obtained from these procedures may be inaccurate. Figure 7 shows that although some wobble was reproducible (e.g., a standard deviation of 0.045 mm), random variations as much as 0.3 to 0.4 mm (at gantry 180° in Fig. 7) were observed at certain gantry or collimator angles. The random variations of the CAX wobble caused uncertainty in the resulting isocenter regardless of which CAX sampling scheme was used. Nevertheless, schemes with larger number of gantry and collimator angles were less sensitive to the random variations of CAX in terms of the isocenter localization accuracy.

The CAX wobble and the resulting isocenter uncertainties were generally small in this study (i.e., on the submillimeter scale). Assessment of such small quantities was feasible by using the digital WL tests. The measurement uncertainty was minimized with the simple phantom setup; the BB was only required to be static during the image acquisition. The image processing was based on a Hough transform algorithm, which located the CAX and the BB with subpixel accuracy. Other computer algorithms capable of subpixel accuracy have also been reported.^30^, ^31^ With these precision tools, we were able to quantify the small errors in isocenter localization due to CAX wobble and different sampling schemes.

There are some limitations in this study. First, all measurements were made on a single type of linac. Although the method can be readily adapted for measurements on other linacs, numerical results from this study may not apply to linacs of different models or manufacturers. Second, we treated all mechanically possible gantry angles and collimator angles equally in CAX sampling. In other words, we assumed that all gantry and collimator angles were used with the same frequencies in clinical treatments. In situations where preference is given to certain gantry or collimator angles, nonuniform weights may be assigned to the CAX samples before the isocenter is determined. As an extreme example, if the patients on a given linac are all treated with collimator angle 0°, then there is no need to sample other collimator angles. Third, we focused on quantifying the CAX wobble and the errors in the resulting isocenters; however, we did not address specifically the causes of these errors. For example, the CAX can wobble due to misalignment of the gantry rotation axis and collimator rotation axis, misalignment of the source position (or beam spot) from the collimator rotation axis, errors in the MLC leaf positions, or the tongue‐and‐groove effect of the MLC. Separation of these effects would be useful for understanding the magnitude of each effect and ultimately finding solutions to minimize these errors. Therefore, future research to systematically investigate the causes of CAX wobble is desirable.

## V. CONCLUSIONS

We investigated the effects of different CAX sampling schemes on the accuracy of WL test‐based isocenter localization. The random errors in isocenter locations were reduced with a large number of CAX samples. The systematic errors arose when inadequate CAX were selected to compensate for the linac mechanical imperfections such as gantry sag, gravitational effect, and collimator misalignment. The systematic errors were reduced by sampling opposing gantry angles and opposing collimator angles. The isocenter localization errors of less than 0.2 mm were achievable by sampling as few as eight CAX (i.e., four cardinal gantry angles and two opposing collimator angles).

## ACKNOWLEDGMENTS

We thank Michael Worley and the Department of Scientific Publications at the University of Texas MD Anderson Cancer Center for editorial review of the manuscript. We are also grateful for the anonymous reviewers' scrutiny of the manuscript and insightful critique.
